# Indoor Particle’s Pollution in Bucharest, Romania

**DOI:** 10.3390/toxics10120757

**Published:** 2022-12-05

**Authors:** Lelia Letitia Popescu, Razvan Stefan Popescu, Tiberiu Catalina

**Affiliations:** 1 Faculty of Building Services, Technical University of Civil Engineering, 021414 Bucharest, Romania; 2 National Institute for Research-Development in Construction, Urbanism and Sustainable Territorial Development—INCD URBAN-INCERC, 400524 Cluj-Napoca, Romania

**Keywords:** PM pollution, experimental, PM_2.5_, PM_10_, PM_2.5_/PM_10_ ratio, rate of change, particles counter, SARS-CoV-2 pandemic period

## Abstract

Air pollution risk factor on human health was surpassed only by high blood pressure, tobacco use and poor diet. Total number of deaths due to air pollution worldwide was estimated to 6.67 million people in 2019. In the European Union, 97% of the urban population is exposed to levels of fine particulate matter above the latest guideline levels set by the World Health Organization. Air pollution accounts for 20% of newborn deaths worldwide, most related to complications of low birth weight and preterm birth. Low birth weight and preterm birth are responsible for 1.8 million deaths worldwide. Bucharest is the capital city of Romania and one of the most polluted cities in Europe, ranking in the 9th position out of 96 of the top cities from Europe and in the 4th position out of 32 of the top cities in Eastern Europe, data from June 2022. The aim of this study was to measure the real time level of indoor particulate pollution levels in different indoor environments from Bucharest, during the pandemic period. The PM_2.5_/PM_10_ ratio and its rate of change were also determined for the measured data. The PM_2.5_/PM_10_ ratio and its rate of change were also calculated based on the measurement data. The PM_2.5_/PM_10_ ratio showed an upward trend on weekends compared to weekdays, suggesting a relationship with outdoor PM where leisure activities and traffic infiltrated the indoors. The fluctuation range of the PM_2.5_/PM_10_ ratio was 0.44~0.95, and low measured values were detected on weekdays. Of the seasons, the proportion of particulate in autumn and its rate of change tended to be higher than in summer. It was suggested that outdoor air may have permeated the room. In addition, the relationship was considered, such as it is a holiday period, there are few rainy days, the concentration of coarse particles is high, and the number of residents in the city decreases. When it comes to indoor air quality, the higher this ratio, the more serious the air pollution. PM_10_ concentrations decreased by 29.1% in the absence of human activity and increased by 35.1% in the presence of humans. PM_2.5_ concentration decreased by 30.3% without human activity and increased by 3.1% with the presence of humans. Certain trends were suggested for the resumption of human activity and an increase in PM_2.5_ concentrations. The average relative difference between October 2021, a pandemic period, and October 2022, a post pandemic period, was 64% for PM_10_ and 47% for PM_2.5_. The pandemic period brought a significantly better indoor air quality from the particulate pollution point of view.

## 1. Introduction

Air pollution was the 4th leading risk factor for early death worldwide in 2019, according to the State of Global Air 2020 Report [1,2,3]. Air pollution risk factor on human health was surpassed only by high blood pressure, tobacco use and poor diet [1]. Total number of deaths due to air pollution worldwide was estimated to 6.67 million people in 2019 [1]. Air pollution accounts for 20% of newborn deaths worldwide, most related to complications of low birth weight and preterm birth. Low birth weight and preterm birth are responsible for 1.8 million deaths worldwide [1,2,3]. The most difficult transition for human lifetime experience is the adaptation from intrauterine to extrauterine life [4]. Particulate matter pollution affects this difficult adaptation for new humans and contributes to almost 500,000 deaths among babies during their first month of life [1]. Outdoor and indoor air pollution is made up of a mixture of airborne particulate matter (PM) and gaseous pollutants [5,6,7,8,9]. Indoor air pollution is due to internal sources [10,11,12,13,14,15], as well as the outdoors as a function of building envelope level of filtration [16,17,18,19,20,21,22]. Outdoor air pollution is made up of a variety of pollutants, generated mainly by vehicle traffic, industrial sources, power plants, agriculture and forest fires [23,24]. Particulate pollutant composition depends on sources of contamination. The source of contamination can be natural or anthropogenic. The particles can be emitted directly into the atmosphere as primary particles or formed in the atmosphere from the transformation of gaseous precursors as secondary particles [25] and photochemical generation of new particles [26,27]. Urbanization and increased population density determined high exposure to traffic-related air pollution, leading to health concerns in both developing and developed countries [28]. The polluted air we breathe is responsible for increasing mortality even in countries known for a lower level of pollution. In 2018, outdoor air pollution caused 84,300 deaths in Italy and 78,400 in Germany, 47,300 in France and 41,900 in the United Kingdom [29]. Agglomerated cities’ road traffic pollution affects a large part of the population. In 2015, it was estimated that 66% of the population in Beijing, 41% in New Delhi, 67% in Paris and 96% in Barcelona were exposed to high levels of pollution due to road traffic [30]. In Sydney, Australia, 16.9% of PM_2.5_ pollution is due to road traffic. The maximum PM concentration in Sydney can reach up to 280 μg/m^3^ [31,32]. Epidemiological studies have shown that coarse (particles measuring between 2.5 to 10 μm in aerodynamic diameter) and fine particles (particles measuring less than 2.5 μm in aerodynamic diameter) and ultrafine particles (particles measuring less than 0.1 μm in aerodynamic diameter) do have large surfaces on which poisonous chemicals, such as metals and PAH (Polycyclic Aromatic Hydrocarbons), viruses (SARS-CoV1,2) can attach and travel [33,34,35]. Ultrafine particles have a higher surface area on which toxic substances can be absorbed, thus being generally considered more toxic for human health than the coarse and the fine [36,37,38]. Fine particles generally represent one to three parts of total particulate matter mass. Within this fraction the ultrafine particles represent a small part of volume but in a large number and thus a large surface area to attach [39]. Both fine and ultrafine particles contain higher concentrations of toxic metals, such as Pb, Cd, and Ni [40], partly because these particles have a higher surface area and volumetric ratio, which give them a more significant potential to transport toxic compounds compared with coarse particles. Fine and ultrafine particles have different toxic species distribution. Fe, Ba, and Cu had more than 70% of their mass in PM_1–2.5,_ whereas Pb, Zn, and Ni showed contributions higher than 60% in the accumulation mode. [41]. Metals and organic components transported by airborne particles cause inflammation of cells, oxidative stress, genotoxicity and even cell death [35,36,37,38,39,40,41,42,43]. In the European Union, 97% of the urban population is exposed to levels of fine particulate matter above the latest guideline levels set by the World Health Organization [44]. Particles penetrate the lungs till the alveolar level, being difficult to be eliminated after [45,46]. The particles with diameters less than 10 µm are considered to be dangerous for the human health. The particles having diameters less than 2.5 µm penetrate the respiratory system till the alveolar level while those with a diameter between 2.5 to 10 µm affect the upper tract of the respiratory system [47,48,49,50,51]. People spend generally most of their time indoors, even more during the pandemic period. Elderly people, children and people with heart or lung disease are more susceptible to inhaling ambient pollutants [23].

Studies on PM_2.5_ and PM_10_ conducted separately are plentiful in the literature, considering their impact on human health, road visibility, climate, and indoor or outdoor air quality. Less research is focused on the relation between PM_2.5_ and PM_10_ given by a ratio with the help of which the different source of particulate matter can be described and locally regulated to reduce emissions, rather than just controlling particulate matter, can be applied [52,53,54,55]. PM_2.5_/PM_10_ ratio was studied for the UK and Arabia [53,54] and for China [55]. Zhao [55] found that PM_2.5_/PM_10_ ratio and its rate of change were high in winter, owing to the low temperature and domestic heating and on weekends with additional leisure activities and traffic travel in a majority of the regions. They concluded that is essential to reduce the PM_2.5_/PM_10_ ratio rather than simply decreasing PM_2.5_. High PM_2.5_/PM_10_ ratios are found in areas with industrial and traffic emissions and low ratios in areas with resuspended dust and sand [55]. For Romania, there are no studies focused on PM_2.5_/PM_10_ ratios, thus our research focused on this parameter.

Annual mean outdoor PM_2.5_ concentration from all measuring stations from Romania for 2018 was 15.10 μg/m^3^ compared with the EU27 annual mean value of 11.59 μg/m^3^ and it caused a number of 21,453 premature deaths [44]. Bucharest is the capital city of Romania and is one of the most polluted cities in Europe, ranking in the 9th position out of 96 in the top cities of Europe and in the 4th position out of 32 in the top cities of Eastern Europe [56], data from June 2022. Indoor air quality is established as a function of outdoor pollution through the building’s envelope, depending on its degree of filtration [16], as well as of the existing internal pollution sources that result from the activity carried out indoors. One of the main sources of pollution in Bucharest is the heavy road traffic in [5], along with the pollution generated by the construction sector. An apparent improvement in air quality in 2020 was observed [44] and is explained by weather patterns and the impact of lockdown measures related to the COVID-19 pandemic period.

In 2021, the PM_10_ annual mean value was 30.77 μg/m^3^ compared with 35.77 μg/m^3^ in 2019, for local station B-3, the closest one regarding our experimental locations, from Romania National Air Quality Network [57]. A total of 22 days with daily average concentration surpassed were declared for 2021 [58].

The aim of this study was to measure the real time level of indoor particulate pollution levels in different indoor environments from Bucharest, during the pandemic period. The outdoor particulate pollution sources impact on indoor air quality, weather and seasons’ influences and indoor activities were considered. The PM_2.5_/PM_10_ ratio and its rate of change were also determined for the measured data.

## 2. Materials and Methods

Indoor air quality measurements from a particulate matter point of view were carried out in Bucharest, in two indoor spaces located on heavy traffic roads. The first measuring point, hereinafter referred to as “Iancului Square”, is an office space located on Mihai Bravu Road, close to Iancului Square. The office space is placed on the 1st floor of a 9 floors building built of reinforced concrete in 1964 and refurnished in 2017, overlooking Mihai Bravu Road, a very crowded street in Bucharest, and Avrig Street, a more quiet small street. In this location, two office rooms were considered during the experiments, one overlooking Mihai Bravu Road, with a floor area of 18 m^2^, named hereafter “Iancului Square (a)” and the second overlooking Avrig Street, with a floor area of 15 m^2^, called “Iancului Square (b)”. Both office rooms are unorganized and naturally ventilated by the occupants. Air conditioning equipment was not used. Only one occupant works in the “Iancului Square (b) office room; three persons work in the other office. The working program was from Monday to Friday, from 9 am.–6 pm. The working program and number of occupants was not affected by COVID-19 restrictions during the experimental campaign, being the same as before. The particles counters were placed on the office table, measuring almost at the nose level of the sitting desk occupant, 1.5 m distance from the windows. During the measurements, the PVC multi-chamber profiles windows were opened or closed; their position will be specified for each case when referring to obtained results. Both rooms contain one laser printer, with a 10 pages per day average use. No coffee machine or carpeted floors are present in the two rooms. The floor is covered with laminar parquet and occupants use the same shoes as outside. The second point of measurements, hereinafter referred to as the “FII Building”, was in the building of the Faculty of Building Services, in an office located on the 2nd floor overlooking Pache Protopopescu Boulevard. The particles counter was placed on the desk, less than 1 m distance from the two rows of old single wooden glaze windows. The office is normally naturally unorganized and ventilated by the professor to whom the office belongs and it is not provided with air conditioning equipment. During the measurements, one of the rows of windows was closed; for the situation in which the rain starts, the second row of windows was opened. During the measuring period, there was no human presence inside the office, neither in the halls nor the offices around, being during the pandemic period and all university activities were taking place online and the university was closed. The floor, with a total area of 8 m^2^, is covered with laminar parquet. There is one coffee machine and one printer, which were not used during the experiments. During the measuring period, the person entered or passed near the office’s door. The building of the Faculty of Building Services is very old and was built in approximately 1900 of burnt clay bricks. The building was first used as a French Catholic girls’ school, which was housed in the building until World War II—“Notre Dame de Sion” Institute. The building was reconsolidated in several stages till approximately 2006 but was not refurnished due to being located in a protected historical area of Bucharest.

**Figure 1:**
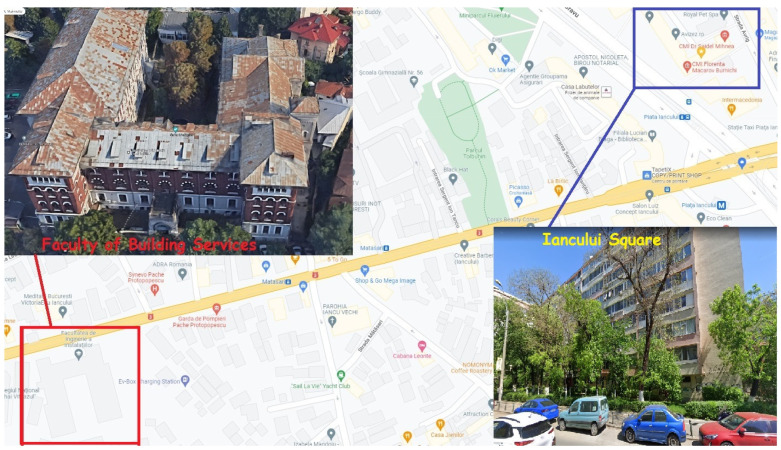
The two measurement locations.

Bucharest ranks in the 8th position of 404 cities considered worldwide, based on traffic index for 2021 [59], with a time lost in traffic per year of 115 h. The first position is occupied by Istanbul, Turkey at 142 h lost per year. A distance of 800 m is between the two measuring points, as the map shows in Figure 1.

**Figure 2:**
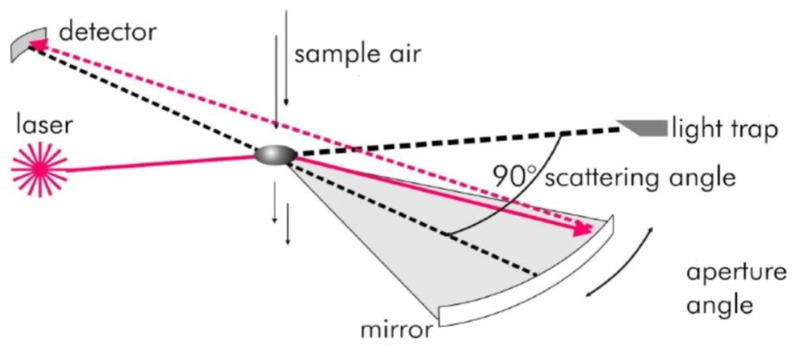
The particles counter principle diagram [Reprinted with permission from [60] 2022].

The particles concentrations were measured with particles counter GRIMM (PLAS—Portable Laser Aerosol Spectrometer, Model Mini-Las 11-E) that has 31 sampling intervals for diameters between 0.25 µm and 32 µm. Optical particles counters use correlation algorithms between the amount of light reflected at an angle θ and the diameter of the particle passing in front of the detector, according to the principle diagram shown in Figure 2 [60]. The operation range for the particles counter is between +4 °C and 40 °C for temperature and relative humidity less than 95%. Particles counter reproducibility is ±3% for the entire measuring range. The particles counter is also provided with a temperature and humidity sensor. The sensor’s temperature span ranges from −40 to 80 °C, with a 0.2 K accuracy. The humidity sensor provides a 0.1% resolution inside the 0 to 100% relative humidity range [61]. Three particles counters, GRIMM 11-E type, were used during the experimental campaign, all of them being just calibrated before the experimental campaign. Real time measurement of dust concentration is therefore possible with data output from 6 s to 60 min. The experimental campaign was conducted from August 2021 till January 2022 and in October 2022. The dust concentration time span was 1 min.

## 3. Results and Discussions

The experimental results are presented hereafter, considering the importance of season of the year, starting from summer till winter, the outdoor sources of pollution impact on indoor air quality, the inside human presence, and the influence of the SARS-CoV-2 pandemic period.

### 3.1. Outdoor Particulate Infiltration, PM_2.5_/PM_10_ Ratio and Its Rate of Change

In order to describe short-term or instantaneous pollution, based on source of pollution description, the rate of change (ROC) for PM_2.5_/PM_10_ is an important parameter to be calculated. This parameter describes the degree of change, meaning that significant ROC implies important changes in emission source type. The formula used for ROC ratio is [55]:

(1) $${\mathrm{ROC}}_{t}=\frac{{\left(\frac{{\mathrm{PM}}_{2.5}}{{\mathrm{PM}}_{10}}\right)}_{t}-{\left(\frac{{\mathrm{PM}}_{2.5}}{{\mathrm{PM}}_{10}}\right)}_{t-1}}{{\left(\frac{{\mathrm{PM}}_{2.5}}{{\mathrm{PM}}_{10}}\right)}_{t-1}}$$

considering ROC_t_ being the rate of change between PM_2.5_ and PM_10_ for the moment t and t − 1 is the time before the moment of the same parameter.

The rate of change (ROC_t_), calculated for the measured data at the “FII Building” location, according to Equation (1) is presented in Table 1. Additionally, the PM_2.5_ and PM_10_ daily average concentrations were calculated, as well as the ratio between the two of them. The experimental campaign was conducted in a period without human activity in the building, as only online courses were scheduled during that period. The office where the measurements were done is located at the 2nd level of the building, meaning almost at 7 m high from the nearby road. The particles counter was place on the desk, less than 1 m away from the closed window, as already presented in the Methods section. As the building was closed, the nearby sources of particles pollution from inside the building can be neglected (e.g., indoor sources of pollution correlated to human activity, PM resuspension, printer, coffee machine, presented in previous section). The outdoor particulate pollution is considered the main source by infiltration through the building envelope, at the window level. The outdoor particulate pollution values were taken from a continuous local measurement station (B-3 point of measurement), the closest situated at a distance of 1140 m from the “FII Building” [57]. Only the outdoor hourly PM_10_ values were available, measured with a Sharp 5030 monitor that combines light scattering photometry and beta radiation attenuation for continuous measurement. The local air quality station, which belongs to Romanian National Network of Air Quality Monitoring, a governmental entity, does not provide PM_2.5_ measured values, and other nearby local stations were not in operation for the needed period of time, in 2021.

Calculated averaged concentrations based on 1 min time span measured values did not exceed daily maximum values for PM_2.5_ (25 µg/m^3^) or for PM_10_ (50 µg/m^3^), according to Romanian Law [62,63]. An indoor PM_10_ daily average concentration of 34 µg/m^3^ (Table 1) at 7 m altitude from the road, measured with closed windows, can signify exceeded levels of pollution at the pedestrian’s level. Indoor PM_10_ daily average values increased until the 8th of October, when they approximately decreased by 38% due to the fact that this day was a rainy one and outdoor coarse particles were washed. The PM_2.5_ daily average concentrations were less influenced by the rain which cleaned the outdoor air, as can be seen in Table 1. An only 10% decrease in the PM_2.5_ daily average concentration appeared after the rainy day.

**Figure 3:**
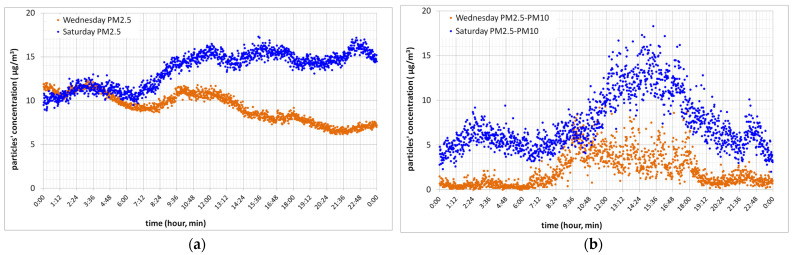
Indoor PM_10_ concentration in time (**a**) and coarse particles’ concentration in time (**b**) for “FII Building” location.

Concerning the PM_2.5_ to PM_10_ ratio, values greater than 0.8 were obtained from Saturday the 2nd of October till Wednesday the 6th of October and again from Sunday the 10th of October till Monday the 11th of October. The ascending trend line of PM_2.5_/PM_10_ ratio starts on Saturday and achieves the maximum value on Sunday. Zhao et al. [55] also found, for outdoor particulate pollution, greater PM_2.5_/PM_10_ ratios values for weekends than weekdays, being correlated to greater private vehicles use during the weekends all day and night, as can be seen in Figure 3. We found this for indoor measured data, but considering the outdoor pollution sources being the main ones.

Outdoor fine particles in the cities are mainly produced by traffic, while the coarse came from industrial and construction sectors, which are closed during the weekend and so affect the PM_2.5_/PM_10_ ratio.

After the weekend, the PM_2.5_/PM_10_ ratio starts to decrease, till the next weekend. The lowest PM_2.5_/PM_10_ ratio was obtained on the 8th of October, just before the rain washed an important amount of outdoor coarse particles. In addition, this day is Friday and PM_2.5_/PM_10_ also increased when the weekend started from the 1st to the 2nd of October. The rate of change highlights the same change in the source of pollution, as the ROC_t_ calculated for weekends is higher than that for weekdays.

**Figure 4:**
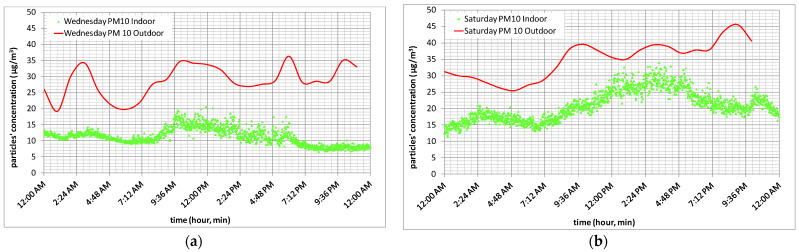
Outdoor and indoor PM_10_ concentration in time for “FII Building” location, for a week-day, Wednesay (**a**) and for a weekend-day, Saturday (**b**).

Figure 3a presents PM_2.5_ concentration in time, and Figure 3b presents particles’, having the mean diameter between 2.5 and 10 µm, concentration in time for site “FII Building”, without human activity, for Wednesday, a weekday, and Saturday, a weekend day. Figure 4 presents outdoor and indoor PM_10_ concentration in time, for Wednesday, a week-day in Figure 4a and for Saturday, a weekend day in Figure 4b, same days considered in Figure 3.

As it can be seen in Figure 3a, PM_2.5_ for Saturday achieves the almost maximum value at approximately 10 a.m. and maintains this level of pollution till 12 a.m. If we consider Wednesday, a working day, high levels of PM_2.5_ appear at approximately 8 a.m. till 2 p.m. So, measured real time particles concentration can be explained as people’s mobility and use of vehicles during weekdays is less than during the weekend, and this represents a reason for which fine particles concentration increases. From 12 a.m. till 7a.m., the Wednesday PM_2.5_ concentration level is approximately the same to Saturday’s PM_2.5_ concentration level. After that moment, the Saturday PM_2.5_ concentration level is 1.5–2 times higher than that of Wednesday. Having the mean diameter between 2.5 and 10 µm, the particles’ concentration increases almost five times for Wednesday during daytime compared to night-time. If we compare Wednesday to Saturday, for the same particles’ diameter span, an increase in their concentration of three to five times can be observed (Figure 3b), which can be due to coarse particles production and accumulation during the interval between the two days, without any rainy day during this period. The increase concentration of particles having the mean diameter between 2.5 and 10 µm from 12 p.m. till 3 p.m. may be attributed to outdoor sporadic work near the measuring point, the resuspension of deposited particles by traffic cars, or an increased wind speed within this time range, which increased outdoor particles through building envelope infiltration, as can be seen in Figure 4a,b where the outdoor PM_10_ ups and downs are exactly followed in the indoor environment, showing again the outdoor particulate infiltration impact on indoor air quality.

**Figure 5:**
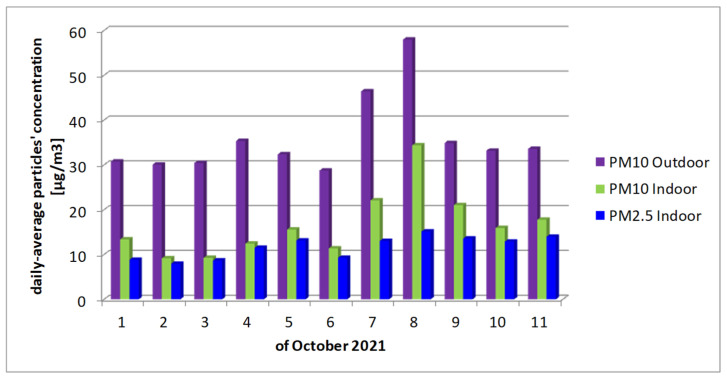
Daily-averaged outdoor PM_10_, indoor PM_10_ and indoor PM_2.5_ for “FII Building”.

Daily-averaged outdoor PM_10_, indoor PM_10_ and indoor PM_2.5_ are compared in Figure 5 for the “FII Building” for the same period of time as in Table 1, from 1 to 11 of October 2021. As already presented, the outdoors particulate pollution values were taken from the closest continuous local air quality station and the indoor values were measured. Figure 5 shows the clear influence of outdoor pollution on indoor pollution; the same trend can be observed for outdoor PM_10_ and the indoor PM_10_ and PM_2.5_, thus showing that outdoor pollution was the main source for indoor pollution for these measurements. A mean value of 0.45 was calculated for the PM_10_ outdoor/indoor penetration factor [16].

**Figure 6:**
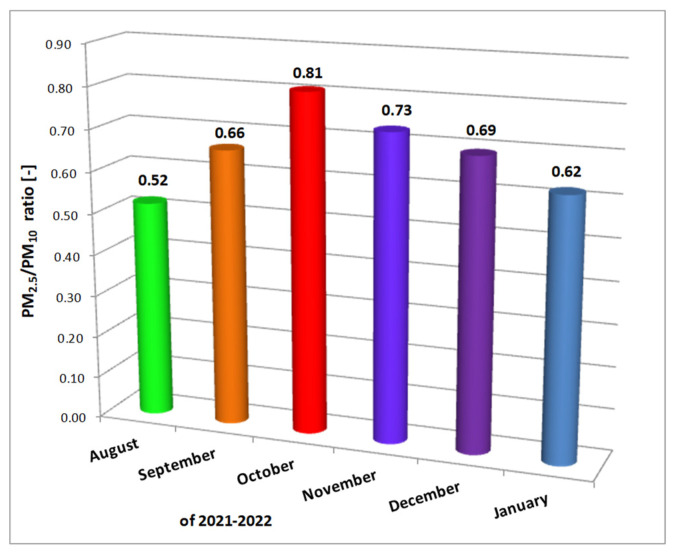
Indoor PM_2.5_/PM_10_ monthly average ratio for “Iancului Square (a)” point of measurement.

Monthly average values for PM_2.5_/PM_10_ ratio calculated for “Iancului Square (a)” site, from August 2021 to January 2022, are presented in Figure 6. As said before, all measurements in “Iancului Square” were also made considering office human activity from Monday to Friday between 9 a.m. to 6 p.m., so indoor and infiltrated outdoor sources determined the measured levels of particulate pollution. Windows were opened during August and September and closed for the other months. The office is unorganized and naturally ventilated by occupants. Indoor sources of pollution, described in the “Methods” section were the same no matter the season. The PM_2.5_/PM_10_ ratio shows an ascending trend from summer to autumn and a descending trend from autumn to winter. During the summer (August, September), the windows were opened, so the outdoor sources influenced more the indoor air quality. If we consider the outdoor sources entered through the opened windows, the summer represents holiday time, with lower road traffic but higher coarse particles levels coming from the construction sector, which is more developed during the summer than during the winter. In September, the schools are opening again and in October also the universities. In September 2021, only elementary educators were present in schools, all the other pupils studied from home in an online format. Yet, even then, the scholars were back from holidays to Bucharest. In October 2021 the Universities started in an online format. The students from outside Bucharest stayed in their original zones as university campuses were mainly closed. In October, the windows were closed, impacting on outdoor PM_10_ infiltration of the indoors. So, lower levels of outdoor PM_10_ could infiltrate through the building envelope than in summer when the windows were opened. Concerning the occupants, the amount of PM_10_ brought on shoes from outside was smaller, as the outside environment was cleaner being washed by rain. The PM_2.5_/PM_10_ ratio difference between the smallest calculated value for August and the highest one for October can also be explained by the fact that in Bucharest in August we barely see the rain, while in October is the opposite. The rain washes the coarse particles, making the fine particles the majority. The decrease of the PM_2.5_/PM_10_ ratio with the winter starts in November and shows that some sectors, being responsible for coarse particles pollution, are starting to close their activity till January, which is the coldest and most snowy month of the year in Bucharest.

Other authors from the literature have observed lower levels of outdoor pollution during the pandemic period [44] than before. Further studies shall be conducted to verify the behavior of pollution levels for our case during and after this period. The next chapter presents a comparison between indoor particulate pollution for October 2021 and October 2022, which highlights the same results as found in the literature.

### 3.2. Human Activity Influence on PM_2.5_/PM_10_ Ratio and Its Rate of Change

Human activity inside the indoor space can modify the PM_2.5_/PM_10_ ratio and the rate of change. In order to highlight the effect of human activity on indoor particulate air pollution, measurements were made in an office space overlooking Avrig Street, at a distance of approximately 100 m from it, called hereafter “Iancului Square (b)” site. We have tried to isolate as far as possible that this can be done in a real office from Bucharest and not in a laboratory; the effect of outdoor particulate pollution sources from the effect of indoor particulate pollution. The main indoor source of particulate pollution for our case was human activity. From the experimental campaign carried out in the “Iancului Square (b)” location, one weekend and the day before and after were considered.

**Figure 7:**
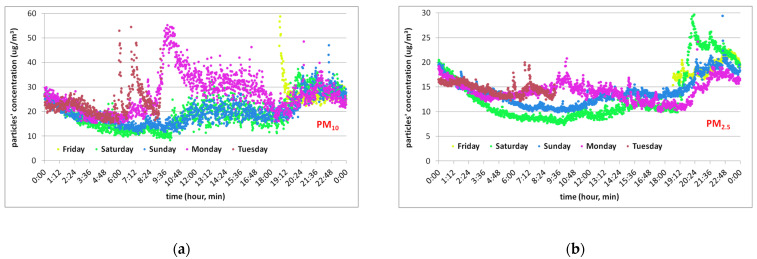
Indoor diurnal variations for PM_10_ (**a**) and PM_2.5_ (**b**) for “Iancului Square (b)” site.

The measurements were performed from Friday to Tuesday, thus including 3 working days (Tuesday was not a full day) and 2 days without activity, from weekend. All measurements were done indoors with the windows closed. For weekdays, human activity was present from 9AM to 6PM, as can be observed in Figure 7. For weekend-days, no human activity indoors was presented. The PM concentrations variation presented in Figure 7 shows the influence of human activity on PM concentration and also outdoor sources’ influence due to the building envelope’s level of filtration.

In Figure 7, the effect of human activity from 9AM to 6PM can be seen on both graphs, for PM_10_ on the left and PM_2.5_ on the right side. Human activity influences re-suspension of PM_10_ more than of PM_2.5_, as shown. Infiltration of outdoor particulate pollution is not obvious during the weekend-days, or for PM_10_ or PM_2.5_, meaning that the particulate variation peaks measured are due only to indoor sources. The variation of the PM_10_ concentration caused by human activity is approximately 29 ± 6% and for PM_2.5_, the decrease in concentration due to lack of activity is approximately 30% but with a very low concentration increase with the resumption of activity of 1 ± 3%. Being in the summer season, coarse particles are more likely brought on the feet from outdoors by occupants and raised in suspension by human activity than by PM_2.5_.

The PM_2.5_/PM_10_ ratio and its rate of change, according to Equation (1) are calculated in Table 2. The rate of change calculated for Monday, with a value of −0.25 compared to 0.05 for Saturday and −0.02 for Sunday (Table 2), shows that the pollution source changed, once human activity resumed. The PM_10_ concentration increased 35% from Sunday to Monday, and only 1% for PM_2.5_ is observed also in the PM_2.5_/PM_10_ ratio calculated for Monday, which is 20.6% lower than that for Sunday.

**Figure 8:**
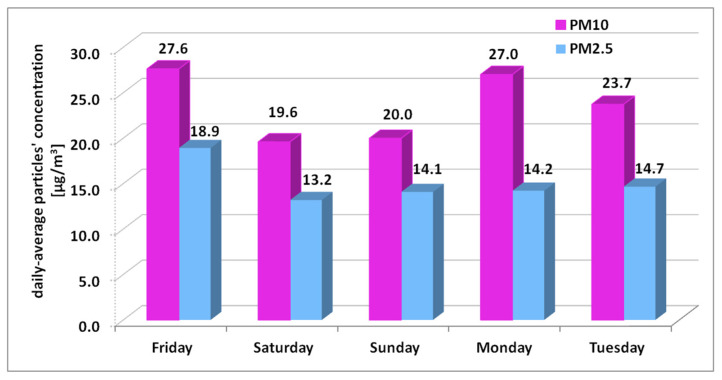
Indoor daily average PM_10_ and PM_2.5_ for weekend-days and days before and after the weekend for “Iancului Square (b)” site.

Daily-average PM_10_ and PM_2.5_ concentrations for “Iancului Square (b)” site are presented in Figure 8. The building unit having an office destination is provided with two rows of new windows on Mihai Bravu Road and one on Avrig Street, with double exposure. The indoor door of the room where the measurements were conducted was always opened, so particulate pollution migration from the other room offices around, where three other persons were working, was allowed. The daily average limits for PM_10_ or PM_2.5_ were not exceeded on any of the days. However, it is worth specifying that the daily average level for particulate pollution varies between 13.2 and 18.9 μg/m^3^ for PM_2.5_ and 19.6 to 27.6 μg/m^3^ for PM_10_, with the windows closed. Figure 8 clearly presents that human activity has great impact indoors on coarse particles concentration, with an almost 30%-30% decrease-increase in concentration without-with human activity. Particle resuspension fractions increase with particle size and walking speed [64].

Indoor PM_2.5_/PM_10_ ratio showed an increased value for weekend-days compared to weekdays, being connected to additional leisure activities and traffic travel, but also to lower activity for industrial and construction sectors. Ratio and its rate of change was higher in autumn than in summer, based on the decrease of the number of inhabitants in the city, being a holiday period. In addition, the autumn brings rainy days, which wash coarse particles, making the fine particles the majority. In terms of indoor air quality, the higher ratio indicated that the air pollution would be more severe. Fine particles (PM_2.5_) are more harmful than large particles, and the higher the PM_2.5_/PM_10_ ratios, the more serious the air pollution. It is important to reduce the proportion of PM_2.5_/PM_10_ rather than just decreasing PM_2.5_. The PM_2.5_/PM_10_ ratio is suitable to be used to mitigate air pollution and improve ambient air quality. Human activity determined an approximately 30% variation in PM_10_ and 30% for PM_2.5_ but with a slower increase with the resumption of activity only for PM_2.5_. The diurnal variations of PM_2.5_/PM_10_ ratios should be further analyzed to better understand the relationship between air pollution and human daily activities.

Indoor PM_2.5_ and PM_10_ calculated daily-average values did not exceed the legal limits, but the experimental campaign took place in 2021–2022, a pandemic period and other studies from the literature have reported lower levels of outdoor pollution during the pandemic period [44], which influence the indoor levels for unorganized naturally ventilated buildings. Further studies are planned for the moment to determine the levels of indoor particulate pollution, as human habits have almost returned to their pre-COVID-19 state.

### 3.3. Indoor Particulate Pollution during the COVID-19 Period and after

Indoor particulate pollution was measured for the “Iancului Square (a)” site, in October 2022 to compare with the data measured in October 2021. In October 2021 the SARS-CoV-2 virus with the so called “Indian variant” was predominant in Bucharest. In October 2021 the total number of new cases of COVID-19 disease in Romania was 414,363, while in September 2022 this number was 49,208 (for October 2022 the data are not available for the moment) [65]. No restrictions related to the virus were applied in October 2022, while those for October 2021 were already presented in this article. All measurements in “Iancului Square (a)” were made also considering office human activity from Monday to Friday from 9 a.m. to 6 p.m., so indoor sources and infiltrated outdoor sources determined the measured levels of particulate pollution. Windows were opened in 2022 and closed in 2021, October 2022 being a warmer period of time. Bucharest’s average outdoor temperature for October 2022 was 14.19 °C, compared with that for October 2021, which was 10.62 °C [66].

**Figure 9:**
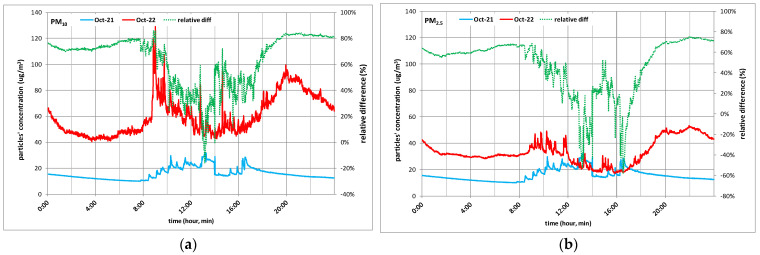
Indoor daily average PM_10_ (**a**) and PM_2.5_ (**b**) for weekend-days and days before and after the weekend for “Iancului Square (b)” site.

Figure 9 presents the indoor monthly average for PM_10_ (Figure 9a) and PM_2.5_ (Figure 9b) for October 2021 and October 2022 and the relative hourly difference between the two of them. As found in the literature for outdoor pollution [44], much higher levels of indoor particulate pollution were found in October 2022 compared to October 2021, as can be seen in Figure 9. The average relative difference between October 2021, a pandemic period, and October 2022, a post pandemic period, was 64%, for PM_10_ and 47%for PM_2.5_. The pandemic period brought a significantly better indoor air quality from a particulate pollution point of view.

## 4. Conclusions

In order to describe particulate matter air pollution, PM_10_ and PM_2.5_ were continuously measured indoors from summer to winter in two location from Bucharest, the Romanian capital, which was in the 4th position of the most polluted cities from Eastern Europe [56], data from June 2022.

The PM_2.5_/PM_10_ ratio and its rate of change were also calculated based on the measurement data. The PM_2.5_/PM_10_ ratio showed an upward trend on weekends compared to weekdays, suggesting a relationship with outdoor PM that infiltrated indoors due to leisure activities and traffic. The fluctuation range of the PM_2.5_/PM_10_ ratio was 0.44~0.95, and low measured values were detected on weekdays.

After a rainy day, the indoor PM_10_ daily average values decreased by 38%, while the PM_2.5_ daily average concentrations were less influenced by the rain and an only 10% decrease in daily average concentration appeared after the rainy day.

Measured real time particles’ concentration can provide a visual presentation of people’s mobility and use of vehicles during a period. For our study, the coarse particles’ concentration increases almost five times for Wednesday during daytime compared to night-time and three to five times for Wednesday to Saturday. This can be due to coarse particles production and accumulation during the interval between the two days, without any rainy day during this period. Of the seasons, the proportion of autumn and its rate of change tended to be higher than in summer. It was suggested that outdoor air may have permeated the room. In addition, the relationship was considered, such as it is a holiday period, there are few rainy days, the concentration of coarse particles is high, and the number of residents in the city decreases. When it comes to indoor air quality, the higher this ratio, the more serious the air pollution. PM_10_ concentrations decreased by 29.1% in the absence of human activity and increased by 35.1% in the presence of humans. PM_2.5_ concentration decreased by 30.3% without human activity and increased by 3.1% with the presence of humans. Certain trends were suggested for the resumption of human activity and an increase in PM_2.5_ concentrations. The average relative difference between October 2021, a pandemic period, and October 2022, a post pandemic period, was 64% for PM_10_ and 47% for PM_2.5_. The pandemic period brought a significantly better indoor air quality from a particulate pollution point of view.

## Figures and Tables

**Table 1:** Indoor PM_2.5_ and PM_10_ daily average concentrations, PM_2.5_/PM_10_ ratio and ROC for “FII Building”.

Measuring Day	Day of the Week	Daily Average Value PM_10_ [µg/m^3^]	Daily Average Value PM_2.5_ [µg/m^3^]	PM_2.5_/PM_10_ Ratio	ROC_t_
1-Oct.-21	Friday	13.4	8.8	0.66	
2-Oct.-21	Saturday	9.2	8.0	0.87	0.32
3-Oct.-21	Sunday	9.3	8.7	0.94	0.08
4-Oct.-21	Monday	12.4	11.5	0.92	−0.01
5-Oct.-21	Tuesday	15.6	13.1	0.84	−0.09
6-Oct.-21	Wednesday	11.4	9.3	0.82	−0.03
7-Oct.-21	Thursday	22.1	13.0	0.59	−0.28
8-Oct.-21	Friday	34.4	15.1	0.44	−0.25
9-Oct.-21	Saturday	21.0	13.6	0.65	0.47
10-Oct.-21	Sunday	15.9	12.9	0.81	0.26
11-Oct.-21	Monday	17.8	14.0	0.79	−0.03

**Table 2:** Indoor PM_10_, PM_2.5_, their ratio and ROC_t_ for weekend days and weekdays for “Iancului Square (b)” site.

Time Span	PM_10_ Variation	PM_2.5_ Variation	ROC_t_	Week-Day	$\frac{PM_{2.5}}{PM_{10}}$
Friday compared to Saturday	29.1%	30.3%		Friday	0.68
Sunday compared to Saturday	2.0%	6.8%	−0.02	Saturday	0.67
Monday compared to Sunday	35.1%	1.0%	0.05	Sunday	0.70
Tuesday compared to Monday	−12.1%	3.1%	−0.25	Monday Tuesday	0.53 0.62

## References

[1] (2020). State of Global Air 2020: A Special Report on Global Exposure to Air Pollution and Its Health Impacts.

[2] Bünger J., Krahl J., Schröder O., Schmidt L., Westphal G.A. (2012). Potential hazards associated with combustion of bio-derived versus petroleum-derived diesel fuel. Crit. Rev. Toxicol..

[3] Sinharay R., Gong J., Barratt B., Ohman-Strickland P., Ernst S., Kelly F.J., Zhang J., Collins P., Cullinan P., Chung K.F. (2018). Respiratory and cardiovascular responses to walking down a traffic-polluted road compared with walking in a traffic-free area in participants aged 60 years and older with chronic lung or heart disease and age-matched healthy controls: A randomised, crossover study. Lancet.

[4] Hillman N.H., Kallapur S.G., Jobe A.H. (2012). Physiology of transition from intrauterine to extrauterine life. Clin. Perinatol..

[5] Popescu R.S., Popescu L. (2017). Assessment of Air Pollution, by the Urban Traffic, in University Campus of Bucharest. J. Environ. Prot..

[6] Ramírez O., de la Campa A.M.S., Amato F., Moreno T., Silva L.F., Jesús D. (2019). Physicochemical Characterization and Sources of the Thoracic Fraction of Road Dust in a Latin American Megacity. Sci. Total Environ..

[7] Rojas J.C., Sánchez N.E., Schneider I., Oliveira M.L., Teixeira E.C., Silva L.F. (2019). Exposure to nanometric pollutants in primary schools: Environmental implications. Urban Clim..

[8] Silva L.F.O., Milanes C., Pinto D., Ramirez O., Lima B.D. (2020). Multiple hazardous elements in nanoparticulate matter from a Caribbean industrialized atmosphere. Chemosphere.

[9] Zamberland D.C., Halmenschelager P.T., Silva L.F.O., da Rocha A., Rocha J.B.T. (2020). Copper decreases associative learning and memory in Drosophila melanogaster. Sci. Total Environ..

[10] Sly P.D. (2021). Adverse Environmental Exposure and Respiratory Health in Children. Pediatr. Clin. N. Am..

[11] Zoran M.A., Savastru R.S., Savastru D.M., Tautan M.N., Baschir L.A., Tenciu D.V. (2021). Exploring the linkage between seasonality of environmental factors and COVID-19 waves in Madrid, Spain. Process Saf. Environ. Prot..

[12] Catalina T., Ghita S.A., Popescu L.L., Popescu R. (2022). Survey and Measurements of Indoor Environmental Quality in Urban/Rural Schools Located in Romania. Int. J. Environ. Res. Public Health.

[13] Mareș I.C., Catalina T., Istrate M.A., Cucoș A., Dicu T., Burghele B.D., Hening K., Popescu L.L., Popescu R.S. (2021). Research on Best Solution for Improving Indoor Air Quality and Reducing Energy Consumption in a High-Risk Radon Dwelling from Romania. Int. J. Environ. Res. Public Health.

[14] Beldean-Galea M.S., Dicu T., Cucoş A., Burghele B.D., Catalina T., Botoş M., Tenter A., Szacsvai K., Lupulescu A., Pap I. (2020). Evaluation of indoor air pollutants in 100 retrofit residential buildings from Romania during cold season. J. Clean. Prod..

[15] Popescu L.L., Popescu R.S. Indoor air measurements for particle pollution. Proceedings of the Conference: 2022 8th International Conference on Energy Efficiency and Agricultural Engineering (EE&AE).

[16] Popescu L., Limam K. (2012). Particle Penetration Research Through Buildings Cracks. HVACR Res..

[17] Popescu L.L. (2009). Influence de la Pollution Exterieure sur la Qualite de l’air Interieur: Role des Fissures dans le Transfert de Particules Solides. Ph.D. Thesis.

[18] Szczepanik-Ścisło N., Scislo L. (2018). Air leakage modelling and its influence on the air quality inside a garage. E3S Web Conf..

[19] Fine J.P., Gray J., Tian X., Touchie M.F. (2020). An investigation of alternative methods for determining envelope airtightness from suite-based testing in multi-unit residential buildings. Energy Build..

[20] Szczepanik-Ścisło N., Schnotale J. CFD simulations and measurements of carbon dioxide transport in a passive house. Refrigeration Science and Technology. Proceedings of the 24th IIR International Congress of Refrigeration, ICR 2015.

[21] Nirvan G., Haghighat F., Wang L.L., Akbari H. (2012). Contaminant transport through the garage—House interface leakage. Build. Environ..

[22] Hajji Y., Bouteraa M., ELCafsi A., Belghith A., Bournot P., Kallel F. (2015). Natural ventilation of hydrogen during a leak in a residential garage. Renew. Sustain. Energy Rev..

[23] Popescu R.S., Popescu L.L. Outdoor air measurements with mobile laboratory in Bucharest. Proceedings of the 2022 8th International Conference on Energy Efficiency and Agricultural Engineering (EE&AE).

[24] Stanek L.W., Brown J.S. (2019). Air Pollution: Sources, Regulation, and Health Effects. Reference Module in Biomedical Sciences.

[25] Kumar P., Wiedensohler A., Birmili W., Quincey P., Hallquist M. (2016). Ultrafine Particles Pollution and Measurements. Compr. Anal. Chem..

[26] de Oliveira Galvao M.F., de Oliveira Alves N., Ferreira P.A., Caumo S., de Castro Vasconcellos P., Artaxo P., de Medeiros S.R.B. (2018). Biomass burning particles in the Brazilian Amazon region: Mutagenic effects of nitro and oxy-PAHs and assessment of health risks. Environ. Pollut..

[27] Chu B., Kerminen V.M., Bianchi F., Yan C., Petäjä T., Kulmala M. (2019). Atmospheric new particle formation in China. Atmos. Chem. Phys..

[28] Miller M.R., Newby D.E. (2020). Air pollution and cardiovascular disease: Car sick. Cardiovasc. Res..

[29] Carvalho H. (2019). Air pollution-related deaths in Europe—Time for action. J. Glob. Health.

[30] Barthelemy J., Sanchez K., Miller M.R., Khreis H. (2020). New Opportunities to Mitigate the Burden of Disease Caused by Traffic Related Air Pollution: Antioxidant-Rich Diets and Supplements. Int. J. Environ. Res. Public Health.

[31] Broome R.A., Powell J., Cope M.E., Morgan G.G. (2020). The mortality effect of PM2.5 sources in the Greater Metropolitan Region of Sydney, Australia. Environ. Int..

[32] Forehead H., Barthelemy J., Arshad B., Verstaevel N., Price O., Perez P. (2020). Traffic exhaust to wildfires: PM2.5 measurements with fixed and portable, low-cost LoRaWAN-connected sensors. PLoS ONE.

[33] (2020). Maladie à Coronavirus (COVID-19): Résumé des Hypotheses.

[34] Kim K.H., Kabir E., Kabir S. (2015). A review on the human health impact of airborne particulate matter. Environ. Int..

[35] Schraufnagel D.E., Balmes J.R., Cowl C.T., De Matteis S., Jung S.H., Mortimer K., Perez-Padilla R., Mice M.B., Riojas-Rodriguez H., Sood A. (2019). Air Pollution and Noncommunicable Diseases: A Review by the Forum of International Respiratory Societies’ Environmental Committee, Part 1: The Damaging Effects of Air Pollution. Chest.

[36] Kwon H.S., Ryu M.H., Carlsten C. (2020). Ultrafine particles: Unique physicochemical properties relevant to health and disease. Exp. Mol. Med..

[37] Phairuang W., Amin M., Hata M., Furuuchi M. (2022). Airborne Nanoparticles (PM0.1) in Southeast Asian Cities: A Review. Sustainability.

[38] Schraufnagel D.E. (2020). The health effects of ultrafine particles. Exp. Mol. Med..

[39] Bhatnagar A. (2019). Air Pollution and Cardiovascular Disease. Braunwald’s Heart Disease: A Textbook of Cardiovascular Medicine.

[40] Liu J.Y., Hsiao T.C., Lee K.Y., Chuang H.C., Cheng T.J., Chuang K.J. (2018). Association of ultrafine particles with cardiopulmonary health among adult subjects in the urban areas of northern Taiwan. Sci. Total Environ..

[41] Rovelli S., Cattaneo A., Nischkauer W., Borghi F., Spinazzè A., Keller M., Campagnplo D., Limbeck A., Cavallo D.M. (2020). Toxic trace metals in size-segregated fine particulate matter: Mass concentration, respiratory deposition, and risk assessment. Environ. Pollut..

[42] Arias-Pérez R.D., Taborda N.A., Gómez D.M., Narvaez J.F., Porras J., Hernandez J.C. (2020). Inflammatory effects of particulate matter air pollution. Environ. Sci. Pollut. Res. Int..

[43] Hernandez M.L., Peden D.B. (2020). Air Pollution: Indoor and Outdoor. Middleton’s Allergy: Principles and Practice.

[44] https://www.eea.europa.eu/publications/air-quality-in-europe-2021/air-quality-status-briefing-2021.

[45] Khomenko S., Cirach M., Pereira-Barboza E., Mueller N., Barrera-Gómez J., Rojas-Rueda D., de Hoogh K., Hoek G., Nieuwenhuijsen M. (2021). Premature mortality due to air pollution in European cities: A health impact assessment. Lancet Planet. Health.

[46] Tanwar V., Adelstein J.M., Wold L.E. (2021). Double trouble: Combined cardiovascular effects of particulate matter exposure and coronavirus disease. Cardiovasc. Res..

[47] El Morabet R. (2018). Effects of Outdoor Air Pollution on Human Health. Reference Module in Earth Systems and Environmental Sciences.

[48] Fattore E., Paiano V., Borgini A., Tittarelli A., Bertoldi M., Crosignani P., Fanelli R. (2011). Human health risk in relation to air quality in two municipalities in an industrialized area of Northern Italy. Environ. Res..

[49] Moreno-Ríos A.L., Tejeda-Benítez L.P., Bustillo-Lecompte C.F. (2022). Sources, characteristics, toxicity, and control of ultrafine particles: An overview. Geosci. Front..

[50] Xiao X., Cao L., Wang R., Shen Z.X., Cao Y.X. (2016). Airborne fine particulate matter alters the expression of endothelin receptors, i.n.rat coronary arteries. Environ. Pollut..

[51] Soppa V.J., Shinnawi S., Hennig F., Sasse B., Hellack B., Kaminski H., Quass U., Schins R.P.F., Kuhlbusch T.A.J., Hoffmanna B. (2019). Effects of short-term exposure to fine and ultrafine particles from indoor sources on arterial stiffness—A randomized sham-controlled exposure study. Int. J. Hyg. Environ. Health.

[52] Zhai X., Mulholland J.A., Russell A.G., Holmes H.A. (2017). Spatial and temporal source apportionment of PM2.5 in Georgia, 2002 to 2013. Atmos. Environ..

[53] Munir S., Habeebullah T.M., Mohammed A.M.F., Morsy E.A., Rehan M., Ali K. (2017). Analysing PM2.5 and its association with PM10 and meteorology in the arid climate of Makkah, Saudi Arabia. Aerosol Air Qual. Res..

[54] Munir S. (2017). Analysing temporal trends in the ratios of PM2.5/PM10 in the UK. Aerosol Air Qual. Res..

[55] Zhao D., Chen H., Yu E., Luo T. (2019). PM2.5/PM10 Ratios in Eight Economic Regions and Their Relationship with Meteorology in China. Adv. Meteorol..

[56] (2022). Pollution Index by City. www.numbeo.com.

[57] https://www.calitateaer.ro/public/home-page/?__locale=ro.

[58] http://www.anpm.ro/documents/12220/2723600/Raport+preliminar+privind+calitatea+aerului+in+Romania_2021.pdf/662d55ac-293f-4e77-8d0f-b2c724b11ceb.

[59] https://www.tomtom.com/traffic-index/bucharest-traffic.

[60] Popescu R.S., Istrate A., Catalina T. (2017). Impact of Ventilation on the Indoor Particulate Matter Concentrations in a School.

[61] (2015). Portable Laser Aerosol Spectrometer. Model Mini-Las 11-E, Manual for Users.

[62] https://legislatie.just.ro/Public/DetaliiDocument/129642.

[63] (2019). Energy Performance of Buildings—Ventilation for Buildings—Part 1: Indoor Environmental Input Parameters for Design and Assessment of Energy Performance of Buildings Addressing Indoor Air Quality, Thermal Environment, Lighting and Acoustics—Module M1-6.

[64] Boulbair A., Benabed A., Janssens B., Limam K., Bosschaerts W. (2022). Numerical study of the human walking-induced fine particles resuspension. Build. Environ..

[65] https://mindcraftstories.ro/coronavirus/evolutia-covid-19-in-romania/.

[66] https://www.wunderground.com/history/monthly/ro/bucharest/LRBS/date/2021-10-14.

